# Principio Obbietto Della Materia Medica

**Published:** 1887-12

**Authors:** 


### Principio Obbietto E Legge Della Materia Medica Omiopatico. Seguita Da un Saggio Di Patogenesia. Del Dottor Tommaso Cigliano. Prezzo Lire 1. Napoli: 1887.

This pamphlet contains an admirable essay upon the "principal objects and laws of the homœopathic materia medica." First, pharmacy, including such topics as triturating, dilutions, etc., is considered; then pharmaco-dynamics; next pharmacology is thoroughly discussed. Lastly is given a repertory of the symptoms of Kali bichromicum. This unique essay was presented by the author to the International Homœopathic Congress held at London in 1881.

